# Considerations for practice-based research: a cross-sectional survey of chiropractic, acupuncture and massage practices

**DOI:** 10.1186/s12906-015-0659-7

**Published:** 2015-05-02

**Authors:** Lysbeth Floden, Amy Howerter, Eva Matthews, Mark Nichter, James K Cunningham, Cheryl Ritenbaugh, Judith S Gordon, Myra L Muramoto

**Affiliations:** Department of Family and Community Medicine, University of Arizona College of Medicine, 1450 N. Cherry Avenue, Tucson, AZ 85719 USA; School of Anthropology, University of Arizona, 1009 E. South Campus Drive, Tucson, AZ 85721 USA

**Keywords:** Complementary and alternative medicine, Practitioners, Chiropractors, Acupuncturists, Massage therapists, Practice-based research, Practice patterns, Cross-sectional survey

## Abstract

**Background:**

Complementary and alternative medicine (CAM) use has steadily increased globally over the past two decades and is increasingly playing a role in the healthcare system in the United States. CAM practice-based effectiveness research requires an understanding of the settings in which CAM practitioners provide services. This paper describes and quantifies practice environment characteristics for a cross-sectional sample of doctors of chiropractic (DCs), licensed acupuncturists (LAcs), and licensed massage therapists (LMTs) in the United States.

**Methods:**

Using a cross-sectional telephone survey of DCs (n = 32), LAcs (n = 70), and LMTs (n = 184) in the Tucson, AZ metropolitan area, we collected data about each location where practitioners work, as well as measures on practitioner and practice characteristics including: patient volume, number of locations where practitioners worked, CAM practitioner types working at each location, and business models of practice.

**Results:**

The majority of practitioners reported having one practice location (93.8% of DCs, 80% of LAcs and 59.8% of LMTs) where they treat patients. Patient volume/week was related to practitioner type; DCs saw 83.13 (SD = 49.29) patients/week, LAcs saw 22.29 (SD = 16.88) patients/week, and LMTs saw 14.21 (SD =10.25) patients per week. Practitioners completed surveys for N = 388 practice locations. Many CAM practices were found to be multidisciplinary and/or have more than one practitioner: 9/35 (25.7%) chiropractic practices, 24/87 (27.6%) acupuncture practices, and 141/266 (53.0%) massage practices. Practice business models across CAM practitioner types were heterogeneous, e.g. sole proprietor, employee, partner, and independent contractor.

**Conclusions:**

CAM practices vary across and within disciplines in ways that can significantly impact design and implementation of practice-based research. CAM research and intervention programs need to be mindful of the heterogeneity of CAM practices in order to create appropriate interventions, study designs, and implementation plans.

## Background

Complementary and alternative medicine (CAM) use has steadily increased globally over the past two decades [1] and is increasingly playing a role in the overall healthcare system in the United States. Use of both self-care and practitioner-based therapies has increased in the past decade [2,3]. US adults report using CAM for primary health care [4], including preventive health [5,6] and treating disease and conditions [5], as well as adjunctive therapy to biomedical care [7-11]. Consistent with this trend has been an uptick in funding for more applied and practice-oriented CAM research, particularly from the National Center for Complementary and Alternative Medicine (NCCAM) [12,13]. As CAM research moves to expand work on practice based effectiveness trials, there is a growing need to better assess and understand the real-world environment of CAM practitioners and their practice. Yet, there is a dearth of published research describing characteristics of real world CAM practices, either in the US or internationally, that could have important impact on the design and conduct of practice-based research and public health interventions.

Previous research on CAM practices has focused on aspects of integrating CAM professional into biomedical practices [14], patient^a^ volume in rural or urban areas [15] or common treatment procedures and reimbursement [16-18]. This information, while helpful for other purposes, does not guide practical considerations for scientists conducting research in these settings. Whether delivering a treatment-specific intervention or a public health intervention across CAM professions, understanding the qualities of how the practitioner and patient interact as well as the functional aspects of the practice setting and environment is a necessary step in the planning phase. Factors such as these can affect the research design and quality of resulting findings [19].

Certain aspects of the practitioner-patient relationship in CAM disciplines have been documented. For example, persons who use CAM services visit their CAM practitioners more frequently than their allopathic physicians [20]. Compared to conventional physicians, CAM practitioners may see patients for longer appointments, [21,22] though chiropractic visit duration can be significantly shorter than that of acupuncture or massage therapy visits (less than 20 minutes for chiropractic vs. 60 minutes for both acupuncture and massage) [23]. Patients expect that CAM practitioners will have a holistic approach [24-26] and provide information and self-help advice [25]. In some cases, CAM practitioners engage patients in discussion of potential treatment outcomes as a way to manage treatment expectations and satisfaction [27]. They are also more likely than conventional allopathic practitioners to use shared decision making for treatment outcomes [28].

Other important aspects of the CAM practice environment have yet to be examined. For example, a critical component for research design is sample size estimation. What is the likely patient volume for each type of CAM practitioner? Sample size estimation (e.g., if a study needs to collect data on 100 patients from each practitioner type) is currently a challenge because it is unclear how many CAM practices would need to be engaged. Also, do CAM practitioners work at one location or multiple sites? Practitioner movement could have a positive implication on design, increasing the potential patient recruitment pool for study participation. However, if the study purpose is to compare interventions between sites, practitioner movement could create a source of data contamination. Can we perform study activities (e.g., recruitment, surveys) in the practitioner office? The way in which CAM practitioners administratively organize their practices and their workflow to deliver services can be quite different than conventional biomedical practitioners. For example, is there administrative staff to interact with patients while waiting for treatment or to provide additional patient care support? Additionally, shared space between independent practitioners may affect how and where study activities can be conducted, particularly if some are involved in the research and others are not.

In this paper, we present findings from a practitioner and practice organization survey that was administered to doctors of chiropractic (DCs), licensed acupuncturists (LAcs), and licensed massage therapists (LMTs) in Pima County, Arizona. We selected these CAM types because they are the most commonly used provider-based CAM therapies in the US [3]. The survey was conducted as part of the CAM Reach study, a NIH-funded practice-based research study of a tobacco cessation brief intervention training and practice system intervention for chiropractors, acupuncturists and massage therapists (CAM practitioners). We present the practitioner and practice organization data through two different lenses: one focusing on the characteristics of practitioners, regardless of their practice composition, and the other focusing on practice location and composition. We discuss the implications of these findings for the CAM Reach study and for future research and programming in the CAM practice context.

## Methods

### Participants, recruitment and informed consent

We obtained names and contact information of practitioners licensed in the state of Arizona with a Tucson, Arizona address from the state licensing boards of each of the three professions: chiropractic, acupuncture and massage therapy. Although licensing requirements vary by state in the US, it is mandatory that all practicing chiropractors, acupuncturists and massage therapists in Arizona maintain a current license. We sent an introduction letter using standard mail to potential participants. The letter described the survey purpose and procedure and advised that a research team member would contact them by telephone within 7–10 days to invite their participation and conduct surveys with interested individuals.

Practitioners were eligible if they were currently practicing in their profession, practiced within the Tucson, Arizona metropolitan area, were over 18 years of age, and were willing to complete a 10–15 minute telephone survey.

If practitioners were interested and eligible, the research staff member read the study consent disclosure aloud, answered any questions and received oral consent to continue with the survey. The University of Arizona Human Subjects Committee approved the study.

### Survey implementation

We administered the survey to LAcs in July 2011, to LMTs in July-August, 2012, and DCs in January-February, 2013 using computer-assisted telephone interviewing software, and recorded responses electronically. All respondents were entered into a raffle to win one of three $50 raffle prizes, which were drawn at the end of the survey period - one for each CAM practitioner type.

### Survey instrument/measures

To best understand how a CAM practice is defined, we designed the survey to identify each location where they delivered services and asked each practitioner to report characteristics of the location and the patient volume associated with each location. Practitioners were queried about solo vs. group locations, whether the location was home-based, what other practitioner-types were associated with the location, if administrative support was available at the location, and how many new and existing patients they treated at each location per week. Because a practitioner may operate at multiple locations, they were asked to complete a survey for each location.

### Analysis

We present findings for two units of analysis, practitioner and location, because a single practitioner could have completed multiple surveys. We calculated a practitioner’s overall patient volume and new patient volume by summing the number of patients (or new patients) for all locations where a practitioner works. We present means and standard deviations for continuous data, medians for skewed or non-Gaussian distributed data, and percentages for proportions of respondents who endorsed binary response items. We did not perform comparative statistical analysis across the three CAM professions since there are known fundamental differences and it was not part of the research question. We used a negative binomial regression to describe the relationship of patient volume and whether a practitioner was solo or not. We chose the negative binomial regression because the distribution of patient volume is over-dispersed, specifically; the variance (629.5) is over 25 times the mean (17.6). This regression model controlled for the type of CAM practitioner. We performed the statistical analysis and constructed figures using R version 2.15.1 [29].

## Results

**Figure 1:**
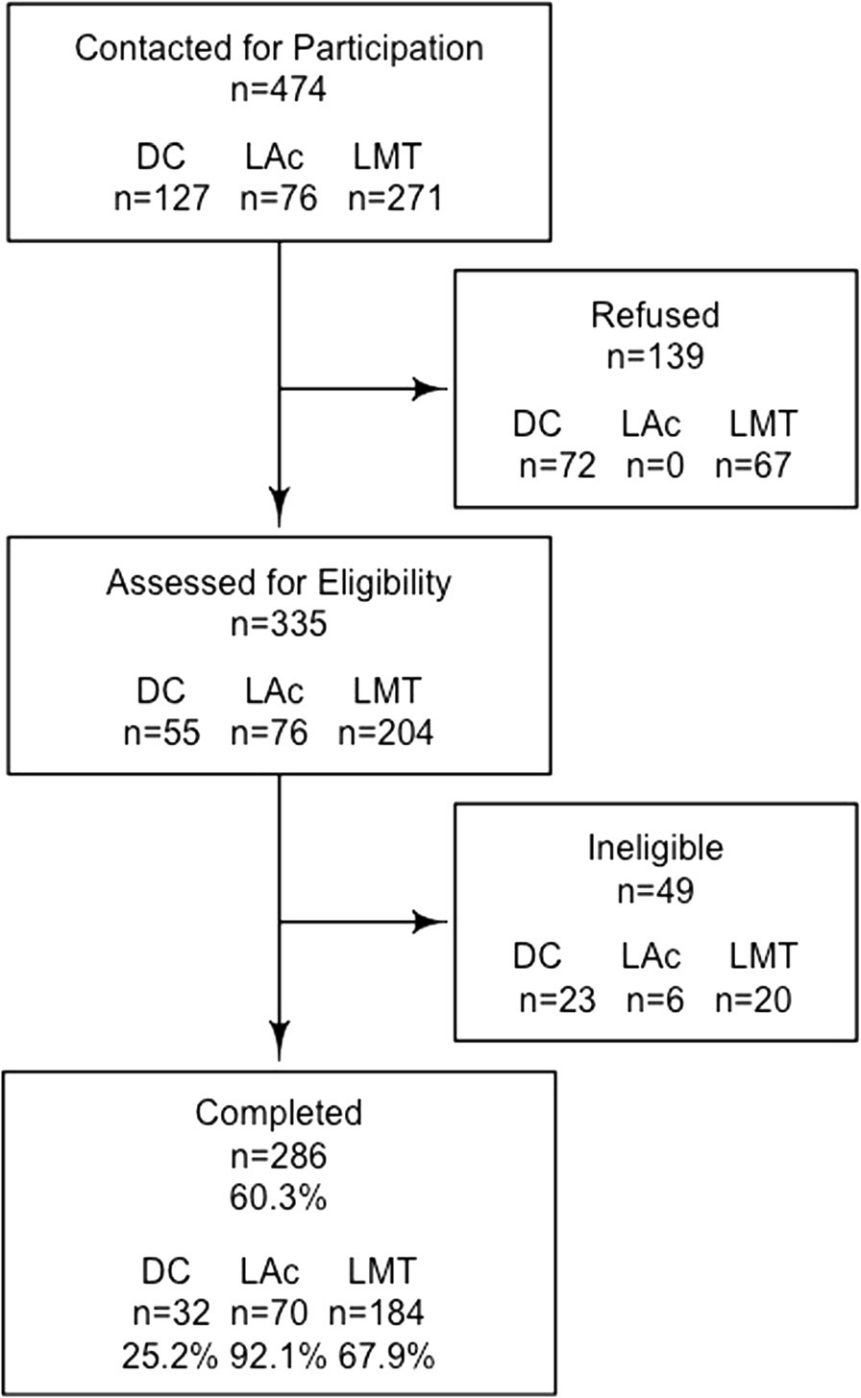
Flow of screening process.

We had contact information for 202 DCs, 123 LAcs and 427 LMTs. Of those, we were able to reach 127 DCs, 76 LAcs and 271 LMTs (i.e. had a correct phone number and answered or replied to study staff telephone calls) within 5 attempts. There were n = 32 DCs, n = 70 LAcs, and n = 184 LMTs who were eligible and consented for a response rate of 25.2% among DCs, 92.1% among LAcs and 67.9% among LMTs (see Figure 1).

### Practitioner considerations

The majority of practitioners reported having only one practice location (93.8% of DCs, 80% of LAcs and 59.8% of LMTs) where they treat patients. A smaller proportion worked in two locations (3.1% of DCs, 17.1% of LAcs, and 36.4% of LMTs), while few practitioners reported working in more than two practices (3.1% DCs, 2.8% LAcs and 3.3% LMTs). When asked where and with whom they practice, practitioners fell into one of five categories. Table 1 depicts these scenarios and the proportions for each profession. Most commonly, practitioners report working in one location as a solo practitioner (44.8% overall, 71.9% of DCs, 58.6% of LAcs and 34.8% of LMTs) or in a group practice (23.8% overall, 21.9% of DCs, 21.4% of LAcs and 25.0% of LMTs). Others practiced at multiple locations: 19.9% of the practitioners worked in at least one solo practice and at least one group practice, 7.0% practiced in multiple group practices, and 4.5% practiced in multiple solo practices. A minority of LAc (17.2%) and LMT (27.9%) practices were home-based. We geographically mapped practice locations, which tended to cluster along major streets/commercial business routes and not within neighborhoods.

**Table 1:** **Practice scenarios**

				
***Single location, solo practice***	***Single location, group practice***	***Multi locations, solo practices***	***Multi locations, solo & group practice***	***Multi locations, group practice***
**n = 128, 44.8%**	**n = 68, 23.8%**	**n = 13, 4.5%**	**n = 57, 19.9%**	**n = 20, 7.0%**
DCs 71.9%	DCs 21.9%	DCs 3.1%	DCs 3.1%	DCs 0.0%
LAcs 58.6%	LAcs 21.4%	LAcs 10.0%	LAcs 7.1%	LAcs 2.9%
LMTs 34.8%	LMTs 25.0%	LMTs 2.7%	LMTs 27.7%	LMTs 9.8%

**Figure 2:**
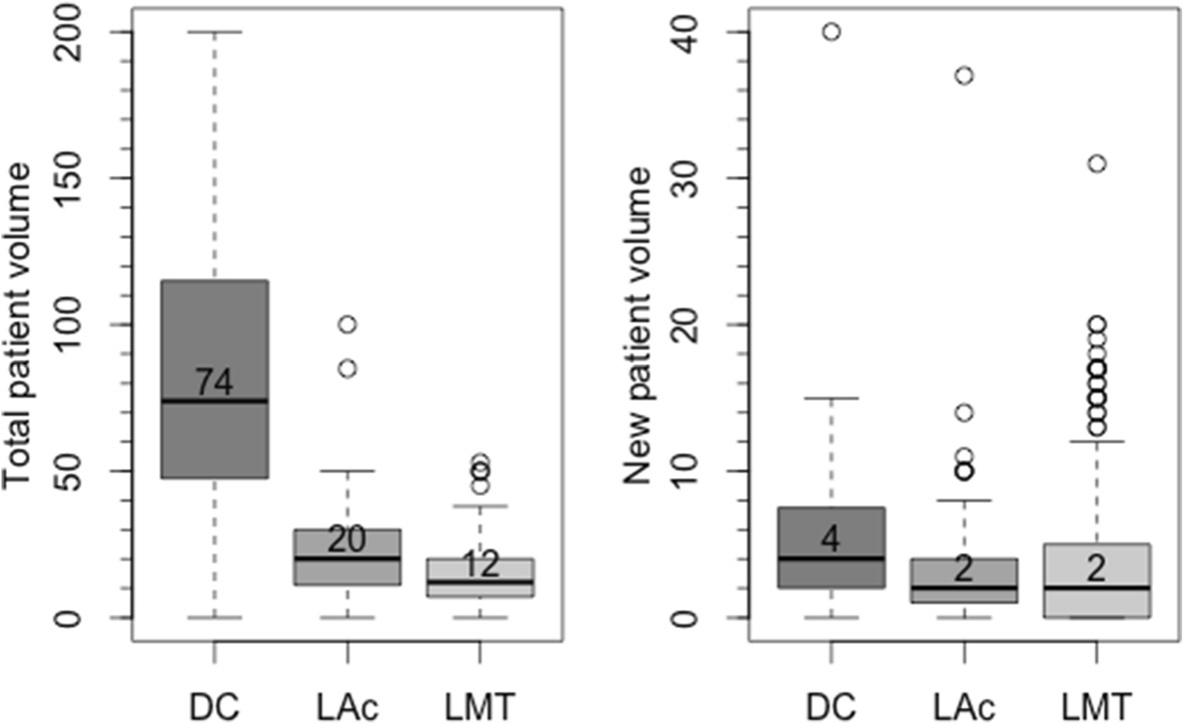
Practitioner weekly total and new patient volume.

The weekly volume of patients was related to the specific CAM discipline and to the type of practice. DCs treated the most patients per week, averaging 83.13 (SD = 49.29, range [0, 200]) patients and 5.69 (SD = 7.23, range [0, 40]) new patients per week. LAcs treated 22.29 (SD =16.88, range [0, 200]) patients and 3.24 (SD = 5.11, range [0, 37]) new patients per week, and LMTs treated 14.21 (SD =10.25, range [0, 53]) patients and 3.85 (SD = 5.50, range [0, 31]) new patients per week. Figure 2 displays the dispersion of patient volume.

### Location considerations

Our practitioners reported information for 388 locations (35 chiropractic locations, 87 acupuncture locations, and 266 massage therapy locations) where they provide treatment. Out of these locations, 9/35 (25.7%) chiropractic practices, 24/87 (27.6%) acupuncture practices, and 141/266 (53.0%) massage practices are composed of more than one practitioner (i.e. are group practices). Negative binomial regression modeling revealed that practitioners at locations where they work alone see fewer patients per week compared to locations where they work with other practitioners (incident rate ratio = 0.70, z = −4.1, p < 0.001, CI [.59, .83]). Table 2 displays characteristics of group practice locations for three CAM disciplines.

**Table 2:** **Composition of group practices**

	**DCs n = 9%**	**n**	**LAcs n = 24%**	**n**	**LMTs n = 141%**	**n**
Practice composition						
- Multiple types of practitioners	33.3	3/9	62.5	15/24	68.8	97/141
- Has administrative staff	88.9	8/9	12.5	3/24	63.8	90/141
- Has DCs	100.0	9/9	20.8	5/24	14.9	21/141
- Has LMTs	22.2	2/9	62.5	15/24	85.1	120/141
- Has LAcs	0.0	0/9	79.2	19/24	18.4	26/141
- Has Naturopaths	0.0	0/9	16.7	4/24	5.0	7/141
- Has Allopathic practitioners	11.1	1/9	8.3	2/24	5.0	7/141
- Has other practitioner types	0.0	0/9	45.8	11/24	57.5	81/141
Business relationship with others in group practice						
- Shares space with other practitioners	11.1	1/9	33.3	8/24	29.8	42/141
- Shares space and administrative staff	11.1	1/9	20.8	5/24	15.6	22/141
- Employee of a group practice	11.1	1/9	16.7	4/24	36.2	51/141
- Owner or Manager of a group practice	33.3	3/9	8.3	2/24	12.8	18/141
- Other type of arrangement	33.3	3/9	20.8	5/24	5.7	8/141

The low percentage of chiropractic practices combined with a small sample size yields a subsample size of n = 9 chiropractic practices that are group practices. This should be noted when making inferences. LMT and LAc group practices more commonly have other types of practitioners than DC group practices (68.8% and 62.5%, compared to 33.3%). All of the DC group practices had practitioners in their same discipline, i.e. had other DCs, compared to 85.1% of group LMT practices that included LMTs and 79.2% of LAc practices that included LAcs. Practitioners of other disciplines and professions were present in 45.8% of LAc and 57.5% of LMT group practices. In the LAc group practices, these included paramedical professionals such as psychologists and physical therapists, and yoga or Pilates instructors, for example. In LMT group practices, these were commonly professionals who work in spa settings such as aestheticians, nail technicians, and hair stylists or paramedical professionals such as physical therapists and nutritionists, for example.

The types of business relationships that practitioners in group practices differed. The most common relationship for LAcs was to share space, or to share space and administrative staff (33.3% and 20.8%, respectively) or another type of arrangement (20.8%). LMTs in a group practice were more commonly an employee of others or sharing space with other practitioners (36.2% and 29.8%, respectively).

## Discussion

The nature and composition of CAM practices is highly variable. To our knowledge, this is the first study in the US or internationally to characterize the variability of patient volume, the number of locations where practitioners work, the types of other practitioners they work with, and the business arrangements that govern how practices function and practitioners interact, and the implications for practice-based research.

The variability of practice characteristics within each CAM discipline is particularly notable. For example, some DCs who consider themselves to be in active practice may not see any patients in some weeks due to seasonal variability in a small, solo practice. Others, who might work in an open access or drop-in visit practice organizational model, may see high patient volume, upwards of 200 patients per week. While the dominant practice scenario for both DCs and LAcs was to work at a single location in either a solo or group practice, there was not a clearly dominant practice model for LMTs: a majority worked at a single location but all practice organization models in Table 1 were well represented. Variability within CAM disciplines needs to be considered when designing interventions and conducting research as this can affect generalizability [19]. Table 3 lists features of CAM practices that may influence research interventions or public health programming. Major considerations differ depending on whether the unit of interest is the whole practice versus the CAM practitioner as an individual.

**Table 3:** **Considerations for CAM research**

**Practice considerations**	**Practitioner considerations**
Patient volume (new vs. return), unique patient visits in a given period of time	Patient volume (new vs. return), unique patient visits in a given period of time
Number and types of practitioners at the location	Business relationship to the practice (e.g. degree of practitioner autonomy)
Number of locations at which practitioners work.	Practitioner payor mix (e.g. self-pay, insurance coverage)
Presence of administrative or clinical support staff for study activities	Time spent at a given location, co-management and referrals of patients between practitioners.

### Practice considerations: patient volume

One characteristic that differentiates these three CAM disciplines is the wide range of total patient volumes. DCs see the most patients by far, as their appointment times are more often shorter in duration than LAcs and LMTs [23]. This high volume potentially means contact with a higher number of total patients, but not necessarily a high volume of new patients or unique patient visits. LAcs and MTs have contact with fewer patients per week, yet have been reported to have greater contact time for each patient [23]. Public health programming or research interventions that target patients will be limited by the number of patients walking through the door at any given practice site.

### Practitioner considerations: patient volume

Conversely, longer appointments could positively impact the type and extent of interventions that could be accomplished in a single visit. Despite the variability in overall patient volume, all three practitioner types had similar volumes of new patients per week (medians 2–4). The ratio of new to return appointments, and the total number of weekly appointments, both have important influences on unique patient volume and on the potential optimal recruitment patterns for research participants.

Of note, when asked to estimate the number of total patients seen at a group practice, practitioners had much difficulty. This is important in the case where one practitioner in a group practice is being called upon by a research project to describe the whole practice. The practitioner’s estimates of practice-wide patient volume may not be reliable.

### Practice considerations: numbers and types of practitioners

The numbers and types of practitioners at a location can significantly impact practice-based research activities or interventions. There is a range of practice types, from large group practices consisting of practitioners from different disciplines that serve as a multidisciplinary CAM practice accommodating a high volume of patients each day, to the single practitioner office that sees only a handful of patients each day. Approximately two-thirds of the group practices where LMTs and LAcs work also had other types of CAM practitioners.

### Practice considerations: number of locations

Over 40% of LMTs work at more than one location (compared to 20% of LAcs and just over 6% of DCs). Practitioner movement between locations can impact practice-based intervention research designs that have a non-intervention group by potentially increasing risks of contamination from the intervention sites. This would have implications for possible ease of patient movement between different practice locations. Additionally, the amount of time a practitioner spends at a location might be an important aspect to factor in to intervention design. A practitioner that works at several locations will have their time divided among locations.

### Practice considerations: presence of administrative staff

Most DC group practices had administrative staff and most LAc group practices did not. Administrative staff who engage the patients upon check-in or checkout are often available to assist in study data collections. They also may have a relationship with repeat patients that make them well suited to explaining study activities to patients. Consequently, practice systems-orientated research that engages auxiliary staff may have more success in DC and, to a lesser extent, LMT group practice settings.

### Practitioner considerations: business relationship

If the intervention focus is on practitioners, the business relationships within a practice can influence their research participation. For example, a single practitioner working as an employee of the practice could have much less autonomy to deliver program interventions or to participate in a research study than a single practitioner working as a independent contractor or solo practitioner/sole proprietor. In a group practice, there may be practitioners who have a solo independent contractor practice and have autonomy at that location, whereas employee practitioners in a group or spa-based practice may not. In our experience with the CAM Reach study, the ability for the practitioners to display and distribute patient education information was affected by the business relationships. Spas, or more commercialized business models, strive to keep a consistent volume and customer experience of the services, and may be less welcoming of practitioners delivering interventions. Practitioners who worked in these settings were limited in the amount of time they had to deliver interventions, in health behavior questions allowed on intake forms, and in the opportunity to distribute patient education materials. On the other hand, practitioners who were not supervised by others, whether in a solo practice or a group practice of independent contractors sharing/renting practice rooms, were able to make small modifications to their usual practices to assess tobacco use in their patients and to offer a brief intervention when appropriate.

### Impact of CAM practice and practitioner characteristics on the CAMR Study

The findings presented in this paper had a direct impact on research study design, implementation, and analysis for the CAM Reach study [30,31]. We found that the way the CAM practice was organized impacted the way in which we could deliver the CAM Reach system intervention and our analytic approach. Most DCs worked at one practice location, it was feasible to enroll all practitioners at the location, and changes from system interventions were applied to the practice as a whole. Consequently, the practice could be the unit of analysis. However, for LMTs and LAcs, the dominant practice characteristics dictated a different approach. Both LAc and LMT practitioners were more likely to work in multiple locations, and work in multiple and more independent business relationships where their practice was not embedded or closely linked within the workflow of other practitioners at that same practice location. They were more likely to work independently of the people sharing space with them, making it necessary to use the *practitioner* as the unit of analysis. While this approach removed a layer of modeling for the statistical analyses, using the practitioner as the unit of analysis also posed some challenges. These practitioners were more likely to work at more than one location so intervention-related changes in the practice environment were more difficult to track. Lastly, some practitioners practice multiple therapeutic areas while primarily identifying themselves as one profession. For example, a chiropractor might also be trained and practice acupuncture or massage therapy. We found this to be important information within the CAM Reach study but outside the scope of this paper.

### Study limitations

This study had limitations. Although it is, to our knowledge, the first study of its kind, to characterize the practitioner composition and the administrative, functional and business, organization of CAM practices either in the United States or abroad, it only included DC, LAc, and LMT practices. These practitioner types are the focus of the CAM Reach study because they are the most commonly visited type of CAM practitioner [2,3]. However there are at least eight other different types of practitioner-based CAM therapies (i.e., Ayurveda, biofeedback, chelation therapy, energy therapy, folk medicine, Homeopathy, hypnosis, and naturopathy) whose practitioners may have other unique aspects to their practice that are relevant to public health researchers and programs. Additionally, the response rate of DCs, in particular, was low. This could pose bias on our sample. For example, it is possible that chiropractors who have higher patient volume might be less likely to respond, for example.

Observations made during data collection of the CAM Reach study included other aspects of CAM practice organization and patient flow that could impact research design or program delivery, although these were not systematically assessed by the survey. For example, some well-established practitioners had practices composed of mostly long-term, repeating visit patients. Others worked in high volume drop-in/open access practices or in spa-based practice where a patient may be seen only once. The Tucson, Arizona location of this study is in the “Sun Belt” with marked seasonal variation in the resident population, and in the use of some types of CAM services and service locations (e.g. day spas associated with resorts). This may have influenced our results in ways that are driven by seasonality. In particular, the DCs were surveyed in the winter months when the resident population of Arizona is at it’s highest level of the year. Increased patient volume may have contributed to the lower response rate from DCs compared to the LAcs and LMTs, who were both surveyed during the summer months. These aspects have the potential to influence programming and research participant recruitment, although this survey did not collect this information. This study did not assess average time spent with patients or the nature of repeat visit patterns.

## Conclusion

CAM practice organizations, practice patterns and patient volumes vary across and within different CAM disciplines in ways that can significantly impact the implementation of both public health and CAM research interventions. As CAM research continues to expand beyond tightly controlled efficacy trials to more practice-based effectiveness trials, it is essential to have a better understanding of how CAM practitioners are organized and function in the real world of CAM practice. Development of practice-based public health programs need to be mindful of the heterogeneity and contexts of CAM practices in order to create appropriate interventions and implementation plans.

## Endnote

^a^Different CAM disciplines customarily use different terms to refer to people seeking their services, i.e. chiropractors and acupuncturists more commonly refer to “patients”, whereas massage therapists refer to “clients.” For simplicity, in this paper we will use “patient.”

## Abbreviations

CAM: Complementary and alternative medicine
NIH: National Institutes of Health
NCCAM: National Center for Complementary and Alternative Medicine
DC: Chiropractor
LAc: Acupuncturist
LMT: Licensed massage therapists
